# Olive oil consumption and all-cause, cardiovascular and cancer mortality in an adult mediterranean population in Spain

**DOI:** 10.3389/fnut.2022.997975

**Published:** 2022-08-30

**Authors:** Laura Torres-Collado, Manuela García-de la Hera, Carla Lopes, Laura María Compañ-Gabucio, Alejandro Oncina-Cánovas, Leyre Notario-Barandiaran, Sandra González-Palacios, Jesús Vioque

**Affiliations:** ^1^Instituto de Investigación Sanitaria y Biomédica de Alicante, ISABIAL-UMH, Alicante, Spain; ^2^Unidad de Epidemiología de la Nutrición, Departamento de Salud Pública, Historia de la Ciencia y Ginecología, Universidad Miguel Hernández (UMH), Alicante, Spain; ^3^CIBER Epidemiología y Salud Pública (CIBERESP), Instituto de Salud Carlos III, Madrid, Spain; ^4^EPIUnit – Instituto de Saúde Pública, Universidade do Porto, Porto, Portugal; ^5^Laboratório para a Investigação Integrativa e Translacional em Saúde Populacional (ITR), Porto, Portugal; ^6^Departamento de Ciências da Saúde Pública e Forenses, e Educação Médica, Faculdade de Medicina, Universidade do Porto, Porto, Portugal

**Keywords:** olive oil, cardiovascular, cancer, all-cause mortality, Mediterranean, nutrition

## Abstract

**Objective:**

We assessed the association between usual olive oil consumption (OOC) and all-cause, cardiovascular (CVD) and cancer mortality in an adult population in Spain.

**Materials and methods:**

OOC was evaluated at baseline in 1,567 participants aged 20 years and older from the Valencia Nutrition Study in Spain using validated food frequency questionnaires. During an 18-year follow-up period, 317 died, 115 due to CVD and 82 due to cancer. Cox regression models were used to estimate adjusted hazard ratios (HR) and 95% confidence intervals (95%CI).

**Results:**

After adjusting for demographic and lifestyle factors, the OOC was associated with a lower risk of all-cause, CVD and cancer mortality. Compared to the less than once per month consumption, the consumption of up to one tablespoon per day was associated with a 9% lower risk of all-cause mortality (HR: 0.91; 95%CI: 0.68-1.22) and the consumption of 2 or more tablespoons with a 31% lower risk of all-cause mortality (HR: 0.69; 95%CI: 0.50–0.93; p-trend = 0.011). The consumption of 2 or more tablespoons per day was also associated with lower risk of mortality for CVD (HR: 0.54; 95%CI: 0.32–0.91; p-trend = 0.018) and cancer (HR: 0.49, 95%CI: 0.26–0.94; p-trend = 0.019).

**Conclusion:**

Higher olive oil consumption was associated with lower long-term risk of all-cause, CVD and cancer mortality in an adult Mediterranean population. The maximum benefit was observed for the consumption of two or more tablespoons per day.

## Introduction

Olive oil consumption (OOC) is highly prevalent in Mediterranean populations, and it has been described as the main source of dietary fat in the Mediterranean Diet (MedDiet). Some recent studies have indicated that OOC is inversely associated with the incidence of cardiovascular complications by reducing the synthesis of interleukin-6, reactive protein-C and other pro-inflammatory molecules (1). There is also evidence from previous studies that habitual OOC can reduce the incidence risk of some chronic diseases such as type 2 diabetes (2), hypertension (3), neurodegenerative diseases (4, 5), stroke or coronary diseases (6–8) some types of cancer (9–11) as well as all-cause and cardiovascular disease (CVD) mortality (12, 13).

In a meta-analysis published in 2014, an inverse association was reported between olive oil consumption (OOC) and the risk of cardiovascular heart diseases (CHD) or stroke (6). However, in this meta-analysis with nine studies including three case-control studies, five prospective cohort studies and one randomized controlled trial from Mediterranean countries (Greece, Italy, France and Spain), an important heterogeneity was reported, and the association was not statistically significant for coronary heart disease (6). In the EPIC cohort study of Spain, participants in the highest quartile of OOC consumption, showed a 26 and 44% reduction for all-cause and CVD mortality, respectively (12). The inverse association between OOC and mortality has also been observed in other populations within the study of dietary patterns. Atkins et al., found that older British men in the highest quartile compared with those in the lowest quartile of OOC had 46 and 37% lower risks of all-cause and CVD mortality (14). In the EPICOR-Study carried out among Italian women, Bendinelli et al. found a significant reduction of 44% in fatal and non-fatal CVD in the highest quartile of OOC (7). In the randomized prevention trial *PREvención con DIeta MEDiterránea* (PREDIMED study), total OOC was associated with 48% reduced risk of CVD mortality although no significant associations were found for all-cause and cancer mortality (13). In the same line, a recently published study conducted in two U.S. population cohorts, the Nurses’ Health Study and the Health Professionals Follow-up Study, showed that compared to those who never or rarely consumed OOC, participants with the highest OOC had a 19% lower all-cause mortality, 19% lower of CVD mortality and 17% lower cancer mortality (15).

Therefore, it seems that the evidence favoring a beneficial effect of OOC on CVD mortality has been increasing during the last few years, whereas the evidence regarding all-cause or cancer mortality is still scarce and not fully consistent, especially in Mediterranean populations. Thus, we aim to evaluate the association between OOC and all-cause, CVD and cancer mortality in an adult Spanish Mediterranean population.

## Materials and methods

### Study design and population

Data were taken from the Valencian Nutrition Survey (VNS), a nutrition and health examination survey conducted in 1994, based on a representative sample from the Valencia Region, Spain (16). Briefly, the VNS recruited 1,811 adults aged 15 years and above between March and July 1994, in the Valencia Region (74.4% participation rate). For the present study, we excluded participants who did not complete the food frequency questionnaire (FFQ) and those younger than 20 years old. Thus, the final sample for this study included complete information for 1,567 participants aged 20 years and above. All participants provided written informed consent. The study was approved by the Ethical Committees of the Hospital of San Juan and the Miguel Hernandez University (DSP.MGH.01.13.).

### Olive oil and dietary assessment

Dietary intake was assessed using a semi-quantitative FFQ at baseline. This FFQ was derived from the Willet questionnaire (17), which was adapted and validated in an adult population in Spain (18). Briefly, the FFQ was validated using four 1-week dietary records in an adult population in Valencia. The validity correlation coefficients (adjusted for energy intake) ranged from 0.27 for folate intake to 0.67 for calcium intake (average 0.47), and the reproducibility correlation coefficient s ranged from 0.30 for carotene intake to 0.65 for calcium intake (average 0.40) (19); this is a similar range to other established diet questionnaires (20).

The FFQ included 93 food items, which comprised the ten main food groups: fruits; vegetables and legumes; dairy; eggs, fish and meat; processed foods; breads and cereals; oils and fats; sweets and pastries and beverages. We asked participants about their usual diet during the previous year, specifically frequency of consumption and standard portion size. For olive oil we questioned on the usual consumption of olive oil added to salads, bread, or foods (1 tablespoon). Frequency of consumption in the FFQ was measured in nine categories, ranging from “never or less than once per month” to “six or more per day.” One item was included to collect the information regarding OOC. We defined OOC using household and standard measures (tablespoon) and we classified subjects according to their frequency of consumption in three categories: never or less than once a month, consumption of ≤1 tablespoon/day, and consumption of 2 or more tablespoons/day. In our FFQ a tablespoon was equivalent to 11 grams of OOC, and the range for categories were non-consumers (0 g/day); ≤1 tablespoon/day (0.7 to ≤11g/day) and ≥2 tablespoon/day (22 to 75.0 g/day).

To estimate the adherence to a traditional MedDiet, we calculated the relative Mediterranean Diet Score (rMED) for each participant (21). This score contained nine components: fruits (including seeds and nuts), vegetables (excluding potatoes), fish, legumes, olive oil and cereals (including whole grain), meat, dairy and alcohol. We excluded olive oil from the score. In the rMED, the intake is calculated in grams for each component (except alcohol), referred to per 1000 kcals/day and divided into tertiles. For the nine categories in the rMED score, we assigned a value of 0, 1, and 2 to the first, second, and third tertiles of intake, respectively. Total meat (including processed meat) and dairy were negatively scored because these components are not representative of the MD (lower scoring for the higher intakes). Alcohol consumption was calculated as a dichotomous variable assigning 2 points for participants with moderate consumption (5–25 g/day for women and 10–50 g/day for men) and 0 points for those with higher or lower consumption. Therefore, we estimated the rMED score for each participant by adding up the points of the 9 components. The rMED ranged from 0 to 6 points (low adherence), 7–10 (medium adherence) to 11–18 points (high adherence). Finally, the information about nutrient values and energy intake were obtained from food composition tables from the United States Department of Agriculture (22) and other Spanish published sources (23).

### Assessment of mortality

The information on the date and cause of death was verified through the National Death Index from the Mortality Registry in the Valencia Region and the Spanish Statistical Office during the 18-year follow-up period.

We used the version 10 of the International Classification of Diseases (ICD-10) to code each cause of death. We created three major categories of deaths as follows: CVD (ICD-10: I00-I99), cancer (ICD-10 codes: C00-D49), and all-cause mortality. All-cause mortality category included deaths from any cause.

### Other variables

Information from socio-demographic characteristics, lifestyles and chronic diseases were obtained from questionnaires completed by personal interviews at baseline. The socio-demographic characteristics considered were: age (in years), sex (men, women), educational level (<primary school; ≥primary school), body mass index (BMI) measured as weight in kilograms divided by the square of measured height in meters (< 25 kg/m^2^, 25–30 kg/m^2^, ≥ 30 kg/m^2^), tobacco smoking habit (never, ex-smoker, current), and total hours of TV watching per day. The presence of pre-existing chronic diseases was also collected such as diabetes (no/yes) and high blood pressure (no/yes). Previous studies have been reported that self-reported diseases in adult population had a high level of agreement in those documented in the medical records (24, 25).

### Statistical analysis

Baseline characteristics of participants were described according to categories of OOC, using means and standard deviation for continuous variables and proportions (number) for categorical variables. Person-years at risk of each subject was estimated from the date of baseline interview to the date of death or the end of follow-up, whichever came first. We evaluated the association between OOC and mortality at 6, 12 and 18 years of follow-up (*ad hoc* cut-off values) to assess the potential short, medium and long-term by estimating adjusted for OOC categories adjusting for potential confounders. We used the Cox proportional hazard regression to calculate hazard ratios (HRs) and their corresponding 95% confidence intervals (95% CI) of all-cause, CVD and cancer mortality using as reference the lower category of OOC (no consumption, ≤ 1 tablespoon/day and ≥ 2 tablespoon/day). The model’s proportional hazard assumption was shown to be adequate based on the scaled Schoenfeld residuals.

The multivariable Cox regression models were adjusted by potential confounders and those variables showing *p* < 0.20 in bivariate analysis: age (in years), sex, educational level, BMI, smoking habit, TV watching, and self-reported diseases (diabetes and hypertension).

We assessed the overall significance of OOC as a categorical variable using the likelihood ratios test (LRT). In addition, a trend test was calculated to explore the dose-response for total OOC, considering the categorical variable as a continuous term.

We also estimated cumulative incidence curves for categories of OOC and all-cause mortality. All analyses were performed using the statistical software STATA, version 16.^1^ All statistical tests were two-sided, and statistical significance was established at *p* < 0.05.

## Results

The baseline characteristics of participants according to OOC are presented in Table 1. Of 1,567 subjects, 18,2% were non-consumers, 44,1% were consumers of ≤ 1 tablespoon/day and 37,7% were consumers of ≥ 2 tablespoon/day. Participants with higher OOC presented higher educational level, lower proportion of diabetes and lower consumption of TV watching.

**TABLE 1:** Socio-demographic and lifestyle characteristics according to olive oil consumption among participants aged 20 years and above of the Valencia Nutrition Study in Spain (*n* = 1567).

	Olive oil consumption				
	Total	None	≤1ts/day	≥2ts/day	*p* ^a^
Participants, *n* (%)	1567 (100.0)	285 (18.2)	691 (44.1)	591 (37.7)	
**Sex, *n* (%)**					
Men	718 (45.8)	125 (43.9)	334 (48.3)	259 (43.8)	
Women	849 (54.2)	160 (56.1)	357 (51.7)	332 (56.2)	0.21
Age, mean (SD)	45.9 (18.0)	47.6 (18.8)	45.4 (18.3)	45.7 (17.4)	0.20
**Education Level, *n* (%)**					
< Primary school	714 (45.6)	158 (55.4)	304 (44.0)	252 (42.6)	
≥ Primary school	853 (54.4)	127 (44.6)	387 (56.0)	339 (57.4)	< 0.001
**Body Mass Index Kg/m^2^, *n* (%)**					
< 25 kg/m^2^	650 (41.7)	101 (35.7)	289 (41.9)	260 (44.3)	
25-30 kg/m^2^	623 (40.0)	116 (41.0)	272 (39.5)	235 (40.0)	
≥ 30 kg/m^2^	286 (18.3)	66 (23.3)	128 (18.6)	92 (15.7)	0.05
**Smoking Status, *n* (%)**					
Never	775 (49.5)	149 (52.3)	339 (49.1)	287 (48.6)	
Ex-smoker	262 (16.7)	42 (14.7)	110 (15.9)	110 (18.6)	
Current	530 (33.8)	94 (33.0)	242 (35.0)	194 (32.8)	0.51
Diabetes ^b^ (yes), *n* (%)	121 (7.7)	34 (11.9)	44 (6.4)	43 (7.3)	0.01
Hypertension ^b^ (yes), *n* (%)	280 (17.9)	57 (20.0)	121 (17.5)	102 (17.3)	0.58
TV watching, hours/day, mean (SD)	2.5 (1.8)	2.8 (1.9)	2.4 (1.8)	2.5 (1.7)	0.02
rMED, mean (SD)	7.2 (2.4)	7.2 (2.4)	7.3 (2.5)	7.2 (2.5)	0.68

VNS, Valencia Nutrition Survey; SD, Standard Deviation; BMI, Body Mass Index; rMED, relative Mediterranean Dietary index; ts: Tablespoon.

^a^ P-value (p) from chi-square test (categorical variables) and ANOVA (continuous variables).

^b^ Self-reported diabetes (no/yes) and hypertension (no/yes).

**FIGURE 1:**
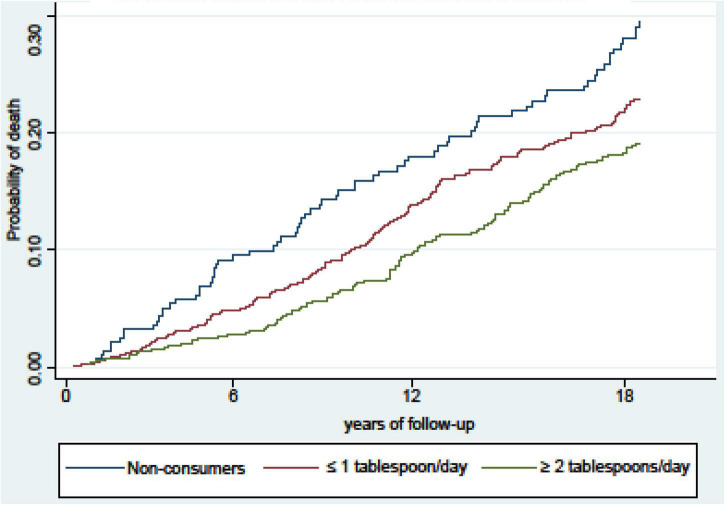
Cumulative incidence curves of death after 18 years of follow-up, according to total olive oil consumption for all-cause mortality in participants from the Valencia Nutritional Survey in Spain.

We documented 85 deaths in the first six years of follow-up (9169.5 person-years) of which 31 were of CVD (36.4%) and 25 were of cancer (29.4%). At 12-years of follow-up (17693.7 person-years), 216 deaths were observed, 77 of which were of CVD (35.6%), and 56 of cancer (25.9%). At the18 years of follow-up (25526.9 person-years), 317 deaths were observed; 115 of which were of CVD (36.3%) and 82 of which were of cancer (25.9%). During the 18-years of follow-up, cumulative incidence curves for all-cause mortality showed that both categories of OOC presented lower risk of mortality than the non-OOC category (Figure 1).

Olive oil consumption (OOC) showed an inverse association with all-cause, CVD and cancer mortality (Table 2). At 6 years of follow-up, compared with non-consumers of olive oil, the consumers of ≥ 2 tablespoon/day showed 60% lower risk of death for all-cause mortality (HR: 0.40; 95% CI: 0.22–0.74), 78% of CVD mortality, (HR: 0.22; 95% CI: 0.07–0.65), and 68% of cancer mortality (HR: 0.32; 95CI%: 0.10–0.96). Similarly, after 12 years of follow-up, we observed 41%, 65%, and 54% lower risk of all-cause, CVD and cancer mortality, respectively. At 18 years of follow-up, we also observed lower risk of all-cause, CVD and cancer mortality among consumers of ≥2 tablespoon/day. Compared to non-OOC, the consumption of ≥2 tablespoon/day, was associated with 31%, 46%, and 51% lower risk of mortality, respectively (Table 2). Significant dose-response trends were observed for categories of OOC and for all types of cause of death (p-trend < 0.05).

**TABLE 2:** Associations between level of olive oil consumption and all-cause, cardiovascular disease and cancer mortality among participants of Valencia Nutrition Survey in Spain (*n* = 1567).

	Olive oil consumption				
	None	≤1ts/day	≥2ts/day	*P*-value^a^	*P*-trend^b^
	**Follow-up at 6 years**				
All-cause (*n*,%)	285 (18.2)	691 (44.1)	591 (37.7)		
Deaths, *n*	27	40	18		
Person-years	1632.9	4044.7	3491.9		
**HR (95% CI)**					
Age and sex adjusted	1.00	0.65 (0.40–1.05)	0.36 (0.20–0.65)	0.003	< 0.001
Multivariable ^c^	1.00	0.69 (0.42–1.16)	0.40 (0.22–0.74)	0.010	0.003
CVD (*n*,%)	270 (17.8)	665 (44.0)	578 (38.2)		
deaths, n	12	14	5		
person-years	1581.9	3966.9	3453.4		
**HR (95% CI)**					
Age and sex adjusted	1.00	0.45 (0.21–0.98)	0.20 (0.07–0.58)	0.002	< 0.001
Multivariable ^c^	1.00	0.45 (0.19–1.06)	0.22 (0.07–0.65)	0.007	0.002
Cancer (*n*,%)	267 (17.7)	662 (43.9)	578 (38.4)		
deaths, *n*	9	11	5		
person-years	1578.0	3941.6	3454.9		
**HR (95% CI)**					
Age and sex adjusted	1.00	0.53 (0.22–1.27)	0.28 (0.09–0.83)	0.061	0.019
Multivariable ^c^	1.00	0.60 (0.24–1.49)	0.32 (0.10–0.96)	0.114	0.037
	**Follow-up at 12 years**				
All-cause (*n*,%)	285 (18.2)	691 (44.1)	591 (37.7)		
deaths, *n*	50	103	63		
person-years	3107.0	7787.3	6799.3		
HR (95% CI)		60			
Age and sex adjusted	1.00	0.85 (0.55–1.19)	0.58 (0.40–0.84)	0.008	0.002
Multivariable ^c^	1.00	0.92 (0.65–1.30)	0.59 (0.40–0.87)	0.001	0.004
CVD (*n*,%)	255 (17.9)	627 (43.1)	546 (38.2)		
deaths, *n*	20	32	18		
person-years	2923.9	7352.6	6476.7		
**HR (95% CI)**					
Age and sex adjusted	1.00	0.72 (0.42–1.24)	0.37 (0.20–0.70)	0.005	0.001
Multivariable ^c^	1.00	0.73 (0.41–1.30)	0.34 (0.17–0.66)	0.003	0.001
Cancer (*n*,%)	247 (17.6)	617 (43.8)	543 (38.6)		
deaths, *n*	12	29	15		
person-years	2879.7	7253.0	6448.7		
**HR (95% CI)**					
Age and sex adjusted	1.00	0.97 (0.49–1.91)	0.46 (0.21–0.99)	0.033	0.025
Multivariable ^c^	1.00	1.08 (0.54–2.16)	0.52 (0.23–1.14)	0.057	0.059
	**Follow-up at 18 years**				
All-cause (*n*,%)	285 (18.2)	691 (44.1)	591 (37.7)		
Deaths, *n*	73	141	103		
Person-years	4462.9	11216.9	9847.4		
**HR (95% CI)**					
Age and sex adjusted	1.00	0.82 (0.56–1.08)	0.64 (0.47–0.86)	0.013	0.003
Multivariable ^c^	1.00	0.91 (0.68–1.22)	0.69 (0.50–0.93)	0.029	0.011
CVD (*n*,%)	243 (17.8)	603 (44.2)	519 (38.0)		
deaths, *n*	31	53	31		
person-years	4089.9	10415.7	9122.7		
**HR (95% CI)**					
Age and sex adjusted	1.00	0.70 (0.45–1.09)	0.48 (0.29–0.80)	0.015	0.003
Multivariable ^c^	1.00	0.89 (0.49–1.26)	0.54 (0.32–0.91)	0.058	0.018
Cancer (*n*,%)	231 (17.3)	590 (44.3)	511 (38.4)		
deaths, *n*	19	40	23		
person-years	3986.5	10262.9	9010.6		
**HR (95% CI)**					
Age and sex adjusted	1.00	0.90 (0.52–1.57)	0.53 (0.29–0.98)	0.061	0.030
Multivariable ^c^	1.00	0.96 (0.54–1.69)	0.49 (0.26–0.94)	0.025	0.019

^a^ p-value from likehood ratio test.

^b^ p-value from p-trend test.

^c^ Cox regression model adjusted for age, sex, educational level (< Primary, ≥Primary school), body mass index (< 25kg/m^2^; 25–30kg/m^2^; > 30kg/m^2^), smoking habit (never; past and current), self-reported diabetes (no/yes), hypertension (no/yes), relative Mediterranean Diet index and TV watching (hours/day).

## Discussion

This study shows that OOC was inversely associated with all-cause, CVD and cancer mortality in a Mediterranean adult population in Spain after 18 years of follow-up. This association was significant for the OOC of two or more tablespoon/day. After the 18-years of follow-up in our study, compared with no-consumption, the consumption of two or more tablespoons per day of olive oil was associated with 31% less risk of all-cause mortality and 46% less risk of CVD mortality. In addition, the consumption of more than one tablespoon per day was also associated with a 51% less risk of cancer mortality.

The association between OOC and mortality presents some controversy. Most evidence supports an inverse association between OOC and all-cause mortality, whereas other studies have shown non-significant associations. For example, in the Greek population of the European Prospective Investigation into Cancer and Nutrition (EPIC-Greek) carried out during 44 months of follow-up, observed no association between OOC and all-cause mortality (26). Similarly, the PREDIMED study, which included 7,216 Spanish participants during 4.8 years of follow-up, did not observe significant associations for all-cause mortality. The findings of these two studies contrast with those described in a meta-analysis based on cohort studies, in which a significant inverse association was found between higher intakes of olive oil and risk of all-cause mortality (RR: 0.77; 95%CI: 0.71–0.84). This inverse association was similar to the observed among the Spanish population in the European Prospective Investigation into Cancer and Nutrition (EPIC-Spain), which reported that participants in the highest quartile of OOC had a 26% lower risk of all-cause mortality in comparison with non-consumers (12). Likewise, in our study, we observed a 31% reduction in all-cause mortality in participants who consumed two or more tablespoons per day during the 18-years of follow-up. The apparent discrepancy among results could be in part due to the different follow-up period in each study, much longer in our study.

Regarding CVD, the OOC was associated with a lower risk of CVD death in our study. We found that participants who consumed two or more tablespoons per day had 46% lower risk of CVD mortality at 18 years of follow-up. This result is similar to that found in the EPICOR study, which observed a reduction in CVD risk among Italian women in the highest quartile of OOC (HR: 0.56; 95%CI: 0.31–0.99) (7), and in the two studies in Spain. In the EPIC-Spain study was reported that subjects in the highest quartile of OOC presented a 44% lower CVD mortality risk (12), and then in the randomized controlled trial PREDIMED study, a 48% lower risk of CVD mortality (13).

Considering cancer mortality, the majority of the studies have reported non-statistically significant associations (12, 13). As far as we know, this is the first study that reported a significant inverse association between OOC and cancer mortality in a Mediterranean country. At 18 years of follow-up, we observed that those who consumed two or more tablespoons per day had 51% lower risk of cancer mortality. Although there is no evidence regarding this association, previous reviews have suggested that OOC could reduce the risk of some specific types of cancer such as breast cancer and upper digestive and respiratory tract neoplasm (27, 28). In a recently published study based on two United States population cohort studies (15), a lower mortality was reported for all-causes, CVD and cancer, thus providing evidence of a potential protective effect of OOC in other non-Mediterranean populations. In any case, more research would be needed to confirm these results in other populations and to explore the effect of OOC by sex and other variables such as smoking and BMI, although we did not observe changes in the estimates other than the loss of significance and some instability due to the low statistical power of subgroup analyses (data not shown).

Previous studies have proposed different mechanisms by which OOC might reduce the risk of mortality. Olive oil is a rich source of MUFAs, vitamin E and several phenolic compounds including oleuropein, hydroxytyrosol and tyrosol, and it has been described biological properties of these compounds including vasodilator, hypoglycemic, antihypertensive, antioxidant and anti-inflammatory effects (29, 30). Hydroxytyrosol has been shown to reduce the formation of atherosclerotic plaques, which increase the risk of coronary heart diseases (30). In addition, hydroxytyrosol promotes the induction of angiogenic genes in hypoxic MCF-7 cells which delays cancer progression (31). Moreover, oleuropein and hydroxytyrosol have been shown to have a protective effect against oxidative damage and inflammatory activity, which are related with diseases such as cancer, diabetes mellitus type 2 and metabolic syndrome (29, 31–33). Thus, olive oil contains several compounds that might have a beneficial effect on health, not only in association with all-cause mortality but also with CVD and cancer mortality.

The current study has several limitations that need to be mentioned. Firstly, we only collected one dietary measurement at baseline, and we were not able to control the changes in OOC during the follow-up. However, as with other consumptions like coffee, OOC might be a dietary habit less susceptible to changes over time (34, 35). In this sense, previous studies have suggested that diet is a habit that remains stable over time and thus, diet assessed at baseline in cohort studies with adult populations might still be a valid method to explore long-term effects on the risk of non-communicable diseases (36, 37). On the other hand, if OOC had changed over time, some non-differential misclassification could be expected, although the bias, if any, would be toward the null, thus reinforcing our findings. Secondly, we did not differentiate between olive oil and extra virgin olive oil consumption, but a previous study showed that most studies do not differentiate between the more widely consumed, “plain” olive oil, and extra-virgin olive oil (28). Lastly, a limitation of this study may relate to the measurement errors from dietary assessment. In our study, OOC was measured as oil added to foods such as salads, bread or foods, without taking into account the oil used in cooking or contained in ready to eat meals. Thus, the total OOC was probably underestimated, although this misclassification should be essentially non-differential.

Strengths of the present study include the well-defined and representative Spanish Mediterranean population, from which we collected information about food intake, socioeconomic characteristics, and lifestyles, using standardized and validated questionnaires. In addition, this study had a long follow-up period that allowed us to detect short and long-term significant associations. Finally, we collected the information regarding OOC before the outcome occurred, thus any misclassification would be non-differential, and could lead to an underestimation of the effects observed in the association between olive oil and mortality, thus reinforcing our results.

## Conclusion

In summary, the present study provides evidence favoring that OOC may be associated with lower all-cause, CVD and cancer mortality in adult Spanish population after a long follow-up period in adult Mediterranean population. Compared with non-consumption, the consumption of 2 or more tablespoon of olive oil per day may reduce the all-cause, cardiovascular and cancer mortality by more than 30%, 40% and 50%, respectively. However, further prospective studies would be warranted to confirm the beneficial effect of OOC and their different types, to make more precise recommendations on olive oil as a component in a healthy food pattern.

## Data availability statement

The raw data supporting the conclusions of this article will be made available by the authors, without undue reservation.

## Ethics statement

The studies involving human participants were reviewed and approved by Ethical approval for the study was given by the Local Ethical Committee of the Hospital of San Juan and the Miguel Hernandez University, Alicante, Spain (DSP.MGH.01.13.). The patients/participants provided their written informed consent to participate in this study.

## Author contributions

LT-C and JV conducted the statistical analyses and drafted the article. All authors revised the article critically for important intellectual content and approved the final version to be published.

## Footnotes

1
https://www.stata.com/
